# The role of city size and urban form in the surface urban heat island

**DOI:** 10.1038/s41598-017-04242-2

**Published:** 2017-07-06

**Authors:** Bin Zhou, Diego Rybski, Jürgen P. Kropp

**Affiliations:** 1 Potsdam Institute for Climate Impact Research (PIK), Member of the Leibniz Association, P.O. Box 60 12 03, Potsdam, D-14412 Germany; 2 Department of Geo- and Environmental Sciences, University of Potsdam, Potsdam, Germany

## Abstract

Urban climate is determined by a variety of factors, whose knowledge can help to attenuate heat stress in the context of ongoing urbanization and climate change. We study the influence of city size and urban form on the Urban Heat Island (UHI) phenomenon in Europe and find a complex interplay between UHI intensity and city size, fractality, and anisometry. Due to correlations among these urban factors, interactions in the multi-linear regression need to be taken into account. We find that among the largest 5,000 cities, the UHI intensity increases with the logarithm of the city size and with the fractal dimension, but decreases with the logarithm of the anisometry. Typically, the size has the strongest influence, followed by the compactness, and the smallest is the influence of the degree to which the cities stretch. Accordingly, from the point of view of UHI alleviation, small, disperse, and stretched cities are preferable. However, such recommendations need to be balanced against e.g. positive agglomeration effects of large cities. Therefore, trade-offs must be made regarding local and global aims.

## Introduction

Urban Heat Island (UHI) is a commonly observed phenomenon worldwide, describing an elevated temperature of urban areas compared to their surroundings. Understanding UHI is of great relevance in the current discussion on sustainable urban design. In particular, heat waves have been observed more persistent and more frequent in the last decades^1, 2^, and are projected to intensify in the future^3^. Furthermore, heat waves are shown to pose an added stress on cities^4^, raising serious concerns regarding general well-being and potential threats to human health, which in turn demands effective adaptation measures to alleviate the UHI.

The UHI effect arises from the anthropogenic modification of natural landscapes and the consequent atmospheric and thermophysical changes in the urban boundary layer^5^. The formation of UHI can be mainly ascribed to an increased absorption and trapping of solar radiation in built-up urban fabrics associated with high thermal admittance of construction materials and the urban canyon structure. Anthropogenic heat release from transport and buildings in the purpose of heating and air conditioning further exacerbate the UHI. Other factors, such as population density, built-up density, and vegetation fractions can also directly or indirectly contribute to the formation of UHI. However, studies based on different spatial scales, more precisely, the vertical scales (urban screen, -canopy, -boundary levels) and the horizontal scales (mirco-, local, regional scales) may lead to varying results on the individual contributions of each factor^6^.

UHI studies can be roughly categorized in two domains regarding the number of investigated cities. On the one hand, case study work focuses on one or a few cities and assesses the UHI characteristics with a high level of detail. On the other hand, ensemble or cross-sectional studies investigate a large number of cities aiming at achieving an understanding of common characteristics or fundamental differences arising among them. The availability of remote sensing surface skin temperature with global coverage has given rise to a number of systematic empirical studies of the latter type. In the following we focus on surface skin temperature and only mention the type explicitly (i.e. surface or 2 m) when necessary.

A global UHI study across more than 400 big cities^7^ revealed that the average annual intensity during daytime is higher than during nighttime and that the daytime intensity correlates negatively with the difference of vegetation cover and activity between urban and suburban areas. Similar diurnal patterns were found in an analysis of 32 Chinese cities^8^. A follow-up work^9^ based on the same Chinese cities suggested an exponential decay of the UHI along urban-rural gradients, the rate and extent exhibit site-specific diurnal and seasonal variations. In Europe, the UHI intensity of urban agglomerations exhibits a size dependency, and can typically reach a maximum of approx. 3 °C in summer and 0.5 °C in winter^10^.

A study^11^ based on 65 cities in North America found that the annual mean daytime and nighttime UHI are positively correlated with the precipitation and the logarithm of population, respectively. It was suggested that the enhanced aerodynamic roughness of densely vegetated rural areas in the humid climate zone (with abundant precipitation) leads to less efficient convection, which hampered the heat transfer from urban to rural areas and resulted in an intensified UHI. This outcome at first glance seems to differ from previous studies^12, 13^ based on air temperature, stating that a deficit of precipitation in the summer leads to stronger rural warming than in urban areas, i.e. a diminished UHI. However, there are substantial differences between these studies besides the data type. The positive correlation in ref. 11 is regressed out of annual mean data (UHI intensity against precipitation) among scores of cities (cross-sectional), whereas the studies^12, 13^ are based on data of individual case study cities across time (temporal).

Another global study comprehensively assessed the dependence of UHI on various urban intrinsic factors, regardless of geographic and climatic factors^14^. Night light, urban area and vegetation are, inter alia, dominant ones accounting for the UHI or urban heat sinks, whereas population and urban structure were found to be of less relevance.

These studies have in common that land cover data is combined with remotely sensed surface skin temperature, i.e. urban land cover is used to define the physical extent of urban areas enabling to systematically extract the temperatures inside the cities and in their rural surroundings. To date, this methodology is an established standard protocol for robustly benchmarking the thermal stress across cities, and for deciphering statistical features of the UHI associated with biophysical and socio-economical indicators. These merits can scarcely be promised by the conventional case study work.

In order to gain an understanding of the UHI phenomenon and its relevance in terms of urban design, insights about the influencing factors are necessary. On the one hand, the UHI intensity of a city is subject to the empirical metrics and indicators used for quantifying the phenomenon^15^. On the other hand, while analyzing its physical essence, it is determined by a variety of factors which can roughly be categorized into (i) external and (ii) intrinsic ones^16^. External factors include location (lat./lon.)^17^, background climate (in particular wind)^10, 18^, proximity to water courses (associated with sea- or lake-breeze circulation), etc., whereas intrinsic ones depict city-specific features (e.g. city size, land cover fractions, anthropogenic heat releases) which, despite being outcomes of long-run urbanization, can be regulated and reshaped.

How to alleviate the UHI effect is another issue of considerable interest. Local interventions (e.g. parks of various sizes, green and cool roofs) are shown to have a limited influence on local climate. The cooling distance, i.e. the maximum distance within which the cooling effect of such green spaces can still be detected, ranges from tens to hundreds meters^19, 20^. Possibilities to influence intrinsic properties – including the overall urban form – are very limited in cities of developed countries due to small growth rates or even negative ones. In contrast, dramatic urbanization is taking place in developing countries, so that insights about how the urban form affects UHI intensities could provide guidance for the large scale planning of cities, where there is a great demand of new infrastructure.

Thus, we search for traceable signatures between features of urban form and UHI intensity. We consider three features of urban form which break down the spatial shape of the urban extent into single values. First, *city size*, since it has been shown previously that larger cities tend to have higher UHI intensities. Second, the *fractal dimension* which represents an established measure to characterize the compactness of a city. Third, *anisometry* which we revealed as an important measure of city shape, quantifies to which extent a city’s length is greater than its width. Examples include cities extending along valleys, rivers, country borders, etc. As we show below, interactions among the three indicators need to be taken into account which implies that the influence of each of them on the UHI intensity cannot be separated.

## Results

Following the methodology employed in previous studies^10, 21^, we combine land cover data with remote sensing temperature data and define the *surface UHI intensity* Δ*T* as the difference between the average temperature within the considered urban cluster and the average temperature within an equal area belt around it (see Methods Section for details). In contrast to ref. 10, here we consider the 5,000 largest urban clusters in Europe and average the summer months (June, July, August) daytime observations from 2006 to 2013. In the following we investigate how the UHI intensity Δ*T* depends on (i) the size, (ii) the fractality, and (iii) the anisometry of the city clusters. Therefore, we need to measure the three quantities for all considered city clusters.

**Figure 1:**
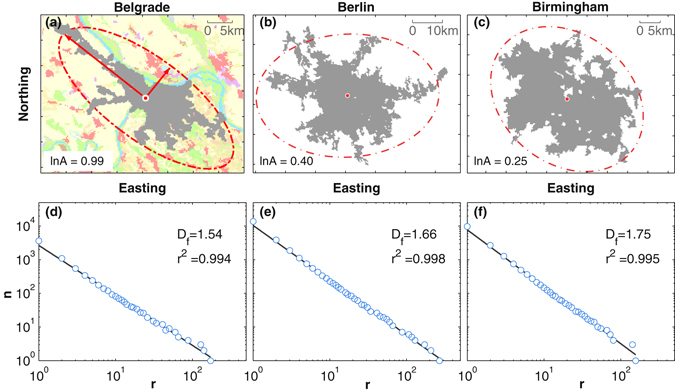
Example of city clusters and box-counting results leading to the fractal dimension (*D*_f_), as well as the logarithm of anisometry (ln *A*). The upper row, panels (**a**–**c**), shows the city clusters as obtained applying CCA to the CORINE land cover data, for the cities of Belgrade (227.56 km^2^), Berlin (854.69 km^2^), and Birmingham (606.38 km^2^). The red ellipse is the equivalent ellipse of the urban cluster, determined by following^50^. The lower row, panels (**d**–**f**), depicts the number of boxes necessary to cover the clusters as a function of the box size in double-logarithmic scale for the corresponding cities. The fractal dimension is obtained from the slope of linear regressions (straight lines). As can be seen from these examples, clusters vary in size, fractal dimension, and anisometry. Esri ArcMap 10.4 (www.esri.com/software/arcgis) and MATLAB R2015b (www.mathworks.com/products/matlab) were used to create the maps.
(iii) The *anisometry A* of a city cluster is defined as the eccentricity of the equivalent ellipse of the city cluster, i.e. the ratio of major axis to its minor axis, see Methods Section. It is a measure for the extent to which the city deviates from an approximate circular shape (*A* → 1), i.e. to which extent it’s length is greater than its width (*A* > 1). Figure 1(a–c) also illustrates the anisometry by means of ellipses. As can be seen, the stretched shape of Belgrade is reflected in a higher value of *A*. Again, we use the logarithm, i.e. ln *A*.

(i) The *city size S*_*C*_ is simply given by the number of cells constituting the city clusters multiplied by the area of each cell, 6.25 × 10^−2^ km^2^. Due to Zipf’s law for cities^22–26^ there are many small cities and few large ones so that we use the logarithm of city size, ln *S*_*C*_, in order to reduce the skewness.
(ii) We compute the *fractal dimension* using the box counting method, assuming $n\sim {r}^{-{D}_{{\rm{f}}}}$, where *n* is the number of (square) boxes of side length *r* necessary to cover the structure, see Methods Section. In Fig. 1(a–c) we show 3 examples of city clusters differing in size and fractality. The corresponding box-counting results for varying *r* are shown in Fig. 1(d–f) and linear regressions in the log-log scale provide the slopes which are an estimate of the fractal dimensions *D*_f_. The fractal dimension *D*_f_ can be considered as a measure of compactness, i.e. compact cities have usually large values of *D*_f_.

**Figure 2:**
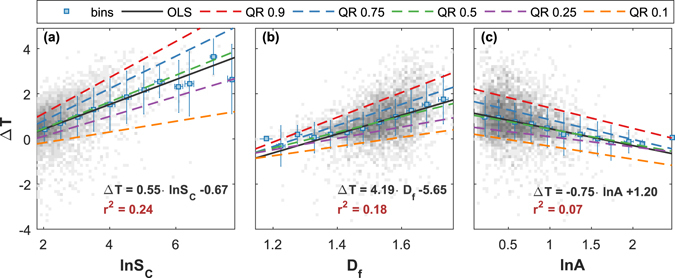
UHI intensity (Δ*T*) as a function of (**a**) logarithm of urban cluster size ln *S*_*C*_, (**b**) fractal dimension *D*_f_, and (**c**) logarithm of anisometry ln *A*, and quantile regressions (QR) as well as ordinary least square regression (OLS). The grey pixels indicate the number of cities that are covered by them (the darker, the higher the density). For visual purpose, the symbols represent averages in equal-width bins and their error-bars represent the standard deviations. The straight lines are linear regressions to raw data, whereas the dashed lines represent the results of quantile regressions. For the quantiles 0.1 and 0.9 we obtained the following slopes: (**a**) [0.24,0.80], (**b**) [2.05, 5.50], (**c**) [−0.58, −0.91].

Figure 2 consists of scatter-plots where the daytime UHI intensity is plotted separately vs. the three quantities – binned values and regressions are included for illustrative purposes. In Fig. 2(a), Δ*T* is displayed as a function of the city size. As expected and consistent with previous work^10, 11, 14, 16, 18, 27^, the UHI intensity increases with city size and doubling the city size leads to approximately 0.4  °C additional UHI intensity. Studies of UHI intensities in relation to population size go back to Oke^28^, who reported both a logarithmic and a power-law (exponent ≈ 1/4) relation between UHI intensity and population. In Fig. 2 we also include quantile regressions and find that there is heteroscedasticity in the form of stronger spreading of Δ*T* among large cities.

In Fig. 2(b) the influence of the fractal dimension on the UHI intensity is shown. In the range where most cities are found, Δ*T* typically increases by roughly 2 °C with increasing *D*_f_. This finding suggests that more compact cities have more pronounced UHI intensities. The literature on UHI intensity and fractality is very limited. The fractal analysis of surface skin temperature related to vegetation abundance^29^ cannot be easily compared with our results since here we study the urban cluster which leads to the UHI. In a more general sense, the influence of urban form has been studied for the example of Beijing metropolitan area^30^, and it has been reported that, compared to a compact city, a dispersed one is efficient in reducing mean urban heat island intensity, but affects the thermal feedback at the regional scale. Last, in Fig. 2(c) Δ*T* is plotted as a function of the anisometry. As one would expect from intuition, the UHI intensity decreases with increasing anisometry, by approximately 1.5 °C in the shown range. Thus, more circular cities seem to exhibit elevated UHI intensities. The above mentioned heteroscedasticity is also observed in Fig. 2(b) and (c), and the spreading of Δ*T* is wider among cities with larger *D*_f_ and smaller ln *A*.

Correlations among the three quantities ln *S*_*C*_, *D*_f_, and ln *A* require a more complex analysis. While anisometry and cluster size are essentially uncorrelated with a Pearson correlation coefficient *ρ* of −0.05 [see supplementary Fig. S2(a)], fractal dimension and anisometry (*ρ* = −0.61) as well as cluster size and fractal dimension (*ρ* = 0.30) exhibit moderate correlations, as shown in supplementary Fig. S2(b) and (c), respectively. On the one hand, cities with lower fractal dimension tend to exhibit higher anisometry, i.e. more compact cities also tend to be more circular. This correlation is the strongest among the three variables considered. On the other hand, larger cities tend to exhibit higher fractal dimensions, i.e. they are more compact. It has been reported previously^31–33^ that city size and fractal dimension are positively correlated, i.e. larger cities in terms of population or urbanized area have higher fractal dimensions.

Thus, we employ multi-linear regression in order to characterize the complex interplay between the UHI intensity and the three factors. Linearity, however, still represents an approximation – but a reasonable one – as will be discussed with the following example. The correlations between UHI intensity and city size *S*_*C*_ have been fitted according to a log-logistic function^10^

1 $${\rm{\Delta }}T({S}_{{\rm{C}}})=\frac{a}{1+{({S}_{{\rm{C}}}/b)}^{-c}},$$

where the parameters *a*, *b*, *c* determine the saturation value, the inflection point, and the steepness, respectively. However, the sigmoid-shape of Equation (1) takes only effect far from the inflection point, i.e. ${S}_{{\rm{C}}}\ll b$ or $b\ll {S}_{{\rm{C}}}$. (i) For small clusters [$\mathrm{ln}({S}_{{\rm{C}}})\to -\infty$] the accuracy of Δ*T* is limited by the resolution of land surface temperature data (≈1 × 1 km^2^). In order to have a reasonable estimate, both cluster and belt temperature should be based at least on a few gridded values. (ii) Due to Zipf’s law for cities (see above), for large clusters [$\mathrm{ln}({S}_{{\rm{C}}})\to \infty$] the sample of cities reduces considerably. As a consequence, there are simply too few data points carrying information on whether or not Δ*T*(*S*_*C*_) saturates. Thus, it is justified to expand Equation (1) in the mid-range. Since around the inflection point the logistic function $F(x)=\mathrm{1/(1}+\exp (-x))$ can be approximated^34^ by $F(x)\approx 1/2+1/4x$, Equation (1) can be approximated by the logarithmic function

2 $${\rm{\Delta }}T({S}_{{\rm{C}}})/a\approx c/4\cdot \,\mathrm{ln}({S}_{{\rm{C}}}/b)+1/2\quad {\rm{for}}\,{S}_{{\rm{C}}}\approx b$$

which corresponds to a linear polynomial of $\mathrm{ln}\,{S}_{{\rm{C}}}$.

After having motivated the *linear* approximation, we finally apply the multi-*linear* regression. In the absence of correlations among the intrinsic urban factors a simple linear combination according to ∆*T* = a + b $\mathrm{ln}\,{S}_{{\rm{C}}}+c{D}_{{\rm{f}}}+d\,\mathrm{ln}\,A$, where *a*, …, *d* are parameters, would be sufficient. Due to the correlations, all interaction terms need to be taken into account. By *interaction* we mean the statistical correlations between two independent variables when multilinearity occurs. We performed a stepwise linear regression with interactions (see Methods Section) to all 5,000 considered city clusters and obtained

3 $${\rm{\Delta }}T=-1.86-0.85\,\,\mathrm{ln}\,{S}_{{\rm{C}}}+1.11\,\,{D}_{{\rm{f}}}+1.45\,\mathrm{ln}\,A+0.83\,{D}_{{\rm{f}}}\,\mathrm{ln}\,{S}_{{\rm{C}}}-1.17\,{D}_{{\rm{f}}}\,\mathrm{ln}\,A$$

with *R* ^2^ = 0.34, all fitting parameters carry the unit °C. According to the analysis, only six out of eight terms contribute statistically to Δ*T*. These are the offset, the three urban factors, and the interaction terms between fractal dimension and size as well as between fractal dimension and anisometry. Consistent with the absence of correlations between size and anisometry (see supplementary Fig. S2a), the corresponding interaction term is missing. Similarly, the three-point-correlation term ${D}_{{\rm{f}}}\,\mathrm{ln}\,{S}_{{\rm{C}}}\,\mathrm{ln}\,A$ statistically does not add information.

As a consequence of the remaining interaction terms, the (linear) dependence of Δ*T* on e.g. *D*_f_ has a varying slope depending on the considered values of ln *S*_*C*_ and ln *A*. For fixed values, e.g. $\mathrm{ln}\,{S}_{{\rm{C}}}=5$ and $\mathrm{ln}\,A=0.5$, Equation (3) simplifies to ${\rm{\Delta }}T({D}_{{\rm{f}}})=-5.38+4.67\,{D}_{{\rm{f}}}$. However, for other values of $\mathrm{ln}\,{S}_{{\rm{C}}}$ and ln *A* both, slope and intercept, are different. A similar effect occurs for ${\rm{\Delta }}T(\mathrm{ln}\,{S}_{{\rm{C}}})$ and ${\rm{\Delta }}T(\mathrm{ln}\,A)$. Due to this complex interplay, it can hardly be visualized in two dimensions how the UHI intensity depends on all of the three intrinsic urban factors.

**Figure 3:**
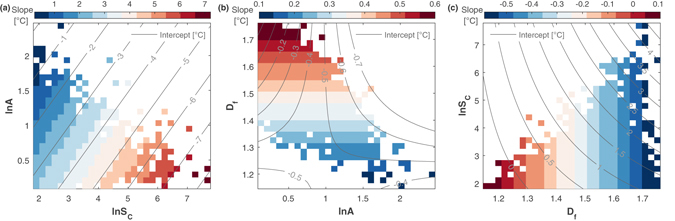
Visualization of Equation (3) as obtained from multi-linear regression for ${\rm{\Delta }}T(\mathrm{ln}\,{S}_{{\rm{C}}},{D}_{{\rm{f}}},\,\mathrm{ln}\,A)$. The panels display the slope (colors) and the intercept (countour lines) of the linear relation between Δ*T* and one urban factor given the other two urban factors are kept constant. For fixed values of ln *S*_*C*_ and ln *A*, Equation (3) simplifies to (**a**) ${\rm{\Delta }}T={\rm{slope}}\cdot {D}_{{\rm{f}}}+{\rm{intercept}}$. Rastering through the relevant ranges of $\mathrm{ln}\,{S}_{{\rm{C}}}$ and ln *A* we show for each combination the corresponding slope and intercept. Analogously, panels (**b**) and (**c**) represent ${\rm{\Delta }}T={\rm{slope}}\cdot \,\mathrm{ln}\,{S}_{{\rm{C}}}+{\rm{intercept}}$ and ${\rm{\Delta }}T={\rm{slope}}\cdot \,\mathrm{ln}\,A+{\rm{intercept}}$, respectively. Combinations which do not occur in the data are kept white. Please note that the range of values covered by the color bar differs among the panels; the figure illustrates the regression, Equation (3) – not the actual data.

Following the above example, Fig. 3 illustrates the linear dependencies of the UHI intensity on one urban factor when the other two are kept constant. Therefore, we fix two of the factors, simplify Equation (3) to a linear form depending only on the third factor, and extract slope and intercept. Then we rasterize the two fixed factors, repeat the procedure, and display the slope and intercept as shown in Fig. 3.

In Fig. 3(a) we observe that Δ*T*(*D*_f_) is steepest for large cities with small anisometry and less steep for small cities with large anisometry. The diagonal stripes are due to the interactions of *D*_f_ with ln *S*_C_ and ln *A*. In Fig. 3(b), ${\rm{\Delta }}T(\mathrm{ln}\,{S}_{{\rm{C}}})$ has its largest slope for compact cities, i.e. large *D*_f_, which only occurs in combination with small anisometry. In this case, the slope only changes along *D*_f_ (horizontal stripes) – interactions with ln *A* have not been found. Lastly, the slope of Δ*T*(ln *A*) is mostly negative [Fig. 3(c)], with the steepest negative slopes observed for cities with a large fractal dimension. The vertical stripes illustrate the interactions with *D*_f_, i.e. there would be no stripes in the absence of interactions.

At this point we still do not know which of the three factors has the strongest influence. The reason is that due to different ranges (e.g. *D*_f_ is roughly within 1.2 and 1.8, while $\mathrm{ln}\,A$ is roughly in the range between 0 and 2.5), the parameters obtained in Equation (3) are not comparable. Thus, we repeat the multi-linear regression, but normalize the data previously to zero mean and unit standard deviation, e.g. ${D}_{{\rm{f}}}^{\ast }=({D}_{{\rm{f}}}-\langle {D}_{{\rm{f}}}\rangle )/{\sigma }_{{D}_{{\rm{f}}}}$ where $\langle {D}_{{\rm{f}}}\rangle$ is the mean and ${\sigma }_{{D}_{{\rm{f}}}}$ the standard deviation. Then we obtain

4 $${\rm{\Delta }}T=0.71+0.33\,\,\mathrm{ln}\,{S}_{{\rm{C}}}^{\ast }+0.23\,\,{D}_{{\rm{f}}}^{\ast }-0.10\,\mathrm{ln}\,{A}^{\ast }+0.06\,{D}_{{\rm{f}}}^{\ast }\,\mathrm{ln}\,{S}_{{\rm{C}}}^{\ast }-0.03\,{D}_{{\rm{f}}}^{\ast }\,\mathrm{ln}\,{A}^{\ast }$$

whereas the 95% confidence interval of the parameters is ±0.03 or smaller. Now we can insert the average values $\langle \,\mathrm{ln}\,{S}_{{\rm{C}}}^{\ast }\rangle$ and $\langle \,\mathrm{ln}\,{A}^{\ast }\rangle$ as typical values (which are both zero due to normalization) and obtain ${\rm{\Delta }}T=0.71+0.23\,{D}_{{\rm{f}}}^{\ast }$. Accordingly, from analogous considerations for the other factors, we see that city size has the strongest influence ($0.33\,\,\mathrm{ln}\,{S}_{{\rm{C}}}^{\ast }$), followed by fractality ($0.23\,{D}_{{\rm{f}}}^{\ast }$), and smallest is the influence of anisometry ($-0.10\,\,\mathrm{ln}\,{A}^{\ast }$). Consistent with Fig. 2, Δ*T* increases with $\mathrm{ln}\,{S}_{{\rm{C}}}^{\ast }$ as well as with ${D}_{{\rm{f}}}^{\ast }$ and decreases with $\mathrm{ln}\,{A}^{\ast }$. However, due to the above discussed interaction, the ranking is only valid for typical cities in our sample and including further small cities could affect the overall outcome. Moreover, we perform a rather basic normalization and we cannot exclude that the skewed distributions could affect the resulting parameters.

Since it has been argued that the UHI intensity based on daytime LST are overestimated^35^, we also included an analysis based on nighttime LST in the Supplementary Information. Due to overall weaker intensities, the dependencies on the city size, fractality, and anisometry are less pronounced. Nevertheless, the relative contributions are consistent with the daytime results.

Certainly, the regression Equations (3) and (4) can hardly capture the huge variations of urban form and heat island intensities for entire Europe. For instance, Berlin (${S}_{{\rm{C}}}=854.69$ km^2^, ${D}_{{\rm{f}}}=1.66$ and $\mathrm{ln}\,A=0.4$) is the largest city among the ones shown in Fig. 1, the measured and predicted temperatures are 3.12 °C and 3.34 °C. For Belgrade (${S}_{{\rm{C}}}=227.56$ km^2^, ${D}_{{\rm{f}}}=1.54$ and $\mathrm{ln}\,A=0.99$) and Birmingham (${S}_{{\rm{C}}}=606.38$ km^2^, ${D}_{{\rm{f}}}=1.75$ and $\mathrm{ln}\,A=0.25$), the measured Δ*T* are 1.39 °C and 3.75 °C; the predicted Δ*T* are 1.82 °C and 3.82 °C, respectively. The three examples suggest that the predictive power of the global regression model, i.e. based on the full sample of cities, is rather limited, which could be due to regional inhomogeneities.

**Figure 4:**
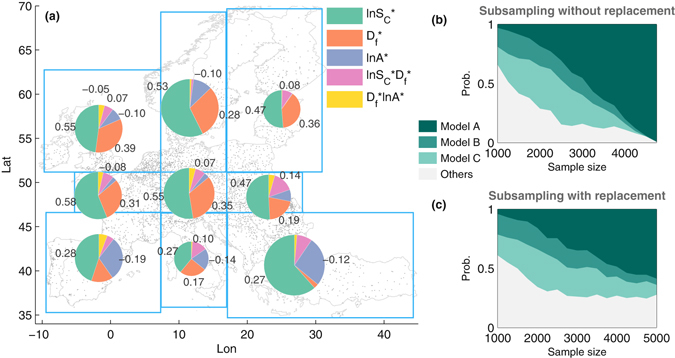
Robustness of multi-linear regression under spatial and random sampling. In panel (**a**) we divide the study area into 9 partitions (blue rectangles) of similar size and separately apply linear regression according to Equation (4) to the normalized quantities. The pie-charts depict the resulting coefficients (for negative values the absolute value has been taken). The area of the pie-charts is proportional to the number of cities in the partition. Only statistically significant coefficients (at 95% level) are labeled. City size dominates in most cases, followed by fractal dimension, whereas in the south the anisometry becomes important. In panels (**b**) and (**c**) the results of stepwise regression on randomly sampled cities (500 repetitions) without and with replacement, respectively, are displayed. In both cases, as the sample size increases, Model A i.e. Equation (4) becomes the most probable model. Model B and C give better estimates under small sample size, and have the forms ${\rm{\Delta }}T\sim \,\mathrm{ln}\,{S}_{{\rm{C}}}+{D}_{{\rm{f}}}+\,\mathrm{ln}\,A+{D}_{{\rm{f}}}\,\mathrm{ln}\,{S}_{{\rm{C}}}$ [i.e. *f*, *g*, $h\simeq 0$ in Equation (6)] and ${\rm{\Delta }}T\sim \,\mathrm{ln}\,{S}_{{\rm{C}}}+{D}_{{\rm{f}}}+\,\mathrm{ln}\,A+\,\mathrm{ln}\,{S}_{{\rm{C}}}\,\mathrm{ln}\,A$ [i.e. *e*, *f*, $h\simeq 0$ in Equation (6)], respectively. MATLAB R2015b was used to create the map (www.mathworks.com/products/matlab).

Therefore, we adopted two different sampling strategies to assess the robustness of the results against the regional inhomogeneities. We first divided the study area into 9 zones of similar number of cities and applied the multi-linear regression in Equation (4), independently. As shown in Fig. 4(a), city size dominates in most cases, followed by fractal dimension, whereas in South Europe anisometry has a larger impact on the UHI. Second, we created subsamples of varying number of cities with and without replacement and applied stepwise regression to the subsamples. Figure 4(b) and (c) reveal that as the sample size increases, our model in Equation (4) tends to appear more frequently. We conclude that our model has a good global performance, while at local scale the model should be used with certain precaution.

## Discussion

In summary, we explore the recently established methodology, which systematically combines urban land cover and remote sensing surface skin temperature, in order to characterize the UHI intensities of a vast number of cities. Studying the largest 5,000 European urban agglomerations, we find a complex interplay among the correlations with intrinsic urban factors. Among the three considered large scale urban features, typically city size has the strongest influence, followed by the fractality – and the anisometry presents the weakest influence. That is, in general, the larger, the more compact (high fractal dimension), and the less stretched (small anisometry) the cities are, the stronger their UHI intensity tends to be.

Our empirical findings on the dependence of the UHI intensity on the city size and form could be attributed to the scale effect of convection^36^. As derived in supplementary, by adopting an idealized urban configuration, the UHI intensity is approximately proportional to ${S}_{{\rm{C}}}^{(1-a{D}_{{\rm{f}}})(1-m)}$ with *a* ≈ 0.43 and $1-m > 0$. For a fixed fractal dimension, as the urban area increases, the heat convection (quantified by the convection heat transfer coefficient *h*) diminishes, resulting in a higher surface temperature. Analogously, for a fixed surface area *S*_*C*_, an increasing fractal dimension *D*_f_ weakens the convection and leads to a higher surface temperature.

Our results can be relevant for urban policy and planning in the context of global warming and local UHI adaptation.

1. Avoid large cities.

How the UHI intensity depends on city size is in particular relevant in world regions of ongoing urbanization. Policies could be developed for incentives to also populate medium size and small cities, i.e. thereby to control the exponent of Zipf’s law for cities^22–26^, which relates the relative frequency of large and small cities.

2. Avoid compact cities.

More compact urban clusters have larger fractal dimensions^37^. Qualitatively, it is comprehensible that urban sprawl and polycentric form lead to smaller fractal dimensions. Urban planning can influence these features of urban form.

3. Avoid rotund cities (i.e. approximately rotational invariance).

It is plausible that stretched cities have lower UHI intensities since the distances to the city border are shorter, in favor of enhanced atmospheric convection. Thus, from an UHI alleviation perspective, cities extending along natural or artificial topographic lines are preferable over those developing mostly around their center.

Certainly, such recommendations need to be opposed to other advantages and disadvantages. In particular, keeping cities small and the consequent ameliorated urban climate should be balanced against positive agglomeration effects of large cities such as shorter trip lengths^38^. Scattered and anisometric cities come along with more traffic, which has negative side effects, including increased anthropogenic heat and CO_2_ emissions. Thus, trade-offs on the *local* scale need to be made, when implementing urban factors. Moreover, from a *global* point of view it has been argued that compact cities are preferred because of their potentials in reducing energy consumption and CO_2_ emissions^39^. However, as mentioned above such recommendations should also be adjusted according to regional specificities (see Fig. 4).

Our work adds to previously gained understanding on how compact urban form increases the UHI intensity and on the problems of transferring such insights into spatial planning^40, 41^. Thus, our results also contribute to the ongoing discussion on the effectiveness of urban forms – in particular, single-centric (compact city) vs. poly-centric city (dispersed city) – as a means for alleviating heat islands as a negative impact of urbanization^30^.

This study is also an example on how concepts from fractal geometry are of use in city science. For three decades, it has been argued that cities are fractal in form^42–44^, and the relation between fractal structures and urban areas has received widespread attention^32^. The fractal dimension of urban agglomerations is a measure of their compactness. Thus, in this study we contribute to the view on cities from a fractals perspective and postulate that the correlations between cluster size and fractal dimension are a manifestation of multi-fractality at the regional scale.

Last but not least, our work opens a perspective for future studies in various directions. First, since here we solely investigate surface skin temperature, an apparent question to be raised is to what extent similar correlations of UHI intensities with urban form also appear considering air temperature. Due to data limitations this can hardly be verified empirically, so that numerical modeling^45^ could represent an alternative. Second, we focus on large scale features of urban form, i.e. intrinsic factors. It could be interesting to test whether the consideration of external factors, foremost wind, would improve the characterization of the influence of intrinsic factors on the UHI intensities. Third, we study ensemble data, i.e. quantify correlations among the sample of cities, and do not consider temporal dynamics. It is important to verify if our findings also hold for an individual city under growth scenarios reflecting the features of urban form^30^. Lastly, can numerical models reproduce our findings or lead at all to comparable results^21^?

## Methods

### Datasets

CORINE urban morphological zones (UMZ) 2006 data at 250 m spatial resolution are used for delineating urban areas in Europe. For countries where raster UMZ data are not available, e.g. UK and Switzerland, we generated the UMZ data based on the CORINE Land Cover 2006 data following the method described in ref. 46. The processed UMZ data, containing binary urban/non-urban information for 38 European countries, are projected to the sinusoidal coordinate system which is consistent with that used in the LST data.

We used the MODIS Aqua 8-day composite (MYD11A2, Version 5) LST products for the summer months (June-July-August, JJA) from 2006 to 2013, in total 104 observations across 8 years. The data are at 926.6 m ≈ 1 km spatial resolution, and are measured at 13:30 (daytime) and 01:30 (nighttime) local solar time. In this study, we focus on the daytime LST, because the daytime surface UHI is more pronounced than that of the nighttime. Complementary results based on nighttime LST can be found in supplementary. According to the pixel-wise LST error flag inherent in MYD11A2, we disregard pixels with LST error >2 K. We also omitted pixels with view zenith angle >35° to minimize the anisotropy bias caused by the view angle, while guaranteeing a sufficient data quantity for further analyses^47^. Based on the processed LST data, multi-annual summer mean LST is calculated.

### Urban heat island (UHI) intensity

We followed the methodology employed in previous studies^7, 10, 21^ to calculate the surface UHI intensity. Cities are defined via the City Clustering Algorithm (CCA)^24, 26, 48^ based on the UMZ data, with a clustering parameter *l* = 250 m, being in accordance with the spatial resolution. According to CCA, any pair of urban cells with a distance no larger than *l* are assigned to the same urban cluster. We defined an equal-area belt region around an identified city cluster as its rural or suburban reference, devoid of water courses and urban pixels of other clusters. The surface UHI intensity of an urban cluster is defined as the difference between average urban and rural temperature^10, 21^, i.e. ${\rm{\Delta }}T={T}_{{\rm{C}}}-{T}_{{\rm{B}}}$. In contrast to previous studies^10, 21^, here we base our analysis on temporally aggregated temperature data, namely multi-annual summer mean LST. Moreover, in contrast to ref. 10, here we disregard small city clusters and consider only the largest 5,000 clusters corresponding to cluster areas *S*_*C*_ > 6.1 km^2^.

### Fractal Dimension

We used the box-counting algorithm to compute the fractal dimension *D*_f_ for each urban cluster^49^. Therefore, we count the number of boxes *N* of size *r* × *r* necessary to fully cover the considered urban cluster. Assuming $N(r)\sim {r}^{-{D}_{{\rm{f}}}}$, the linear regression to $\mathrm{ln}(N)$ vs. ln(*r*) provided the slope which corresponds to the fractal dimension *D*_f_. The conventional method is to initialize *r* to the minimum cell size and stepwise double it until $N(r\mathrm{)=1}$. It turned out that this 2-based exponential sampling method led to a discreteness artefact and denser sampling was more robust. Thus, we adopted a denser sampling strategy by incrementing *r* by 1 and omitting any point [*r*, *N*(*x*)] if the count $N(r)=N(r-\mathrm{1)}$. Sampling can be seen in Fig. 1(d–f).

### Anisometry

We computed the anisometry (*A*) of city clusters similar to the method^50^. We defined the anisometry of a city cluster as the ratio of the city cluster’s maximum Feret’s diameter to its minimum Feret’s diameter. The Feret’s diameter is the distance between two parallels tangent to an object along a certain direction. In order to illustrate the relative stretch of clusters, we drew the equivalent ellipse of a city cluster by assigning the maximum and minimum Feret’s diameters to the axes of the ellipse (see. Figure 1(a–c). The ellipse centers at the centroid of a city cluster. Analog to the cluster size, we use the logarithms of anisometry (ln *A*) throughout the study to reduce the skewness.

### Quantile regression

Quantile regression^51, 52^ is a method for estimating the impact of observed covariates on quantiles of the response variable. In contrast to ordinary least squares regression, quantile regression is particular applicable for the model with heterogeneous variance, e.g. in the presence of heteroscedasticity, where the former approach usually misestimates the real relationship or fails to detect the nonzero changes^53^. Quantile regression finds wide application in disciplines, where data are seldom normally distributed, e.g. ecology^53^, climatology^54, 55^, etc. Assuming a regression function $Y=\beta X+\varepsilon$. The estimators for the quantile *τ*, i.e. ${\beta }_{\tau }$ are obtained by minimizing the sum of asymmetrically weighted absolute residuals. The weights are given by the function ${\rho }_{\tau }$ ^52^.

5 $${\beta }_{\tau }=\arg \,\min \sum _{i\mathrm{=1}}^{n}{\rho }_{\tau }({Y}_{i}-\beta {X}_{i})$$

### Multi-linear regression

We employed the general multi-linear model to quantify the relation between the UHI intensity Δ*T* and predictive variables – the logarithm of city size $\mathrm{ln}\,{S}_{{\rm{C}}}$, fractal dimension *D*_f_, and the logarithm of anisometry $\mathrm{ln}\,A$. We use the general ansatz

6 $${\rm{\Delta }}T=a+b\,\mathrm{ln}\,{S}_{{\rm{C}}}+c\,{D}_{{\rm{f}}}+d\,\mathrm{ln}\,A+e\,{D}_{{\rm{f}}}\,\mathrm{ln}\,{S}_{{\rm{C}}}+f\,{D}_{{\rm{f}}}\,\mathrm{ln}\,A+g\,\mathrm{ln}\,{S}_{{\rm{C}}}\,\mathrm{ln}\,A+h\,{D}_{{\rm{f}}}\,\mathrm{ln}\,{S}_{{\rm{C}}}\,\mathrm{ln}\,A,$$

where *a*, …., *h* are eight parameters, and e.g. *D*_f_ ln *S*_*C*_ is the interaction between fractal dimension and city size. We used the forward and backward stepwise regression to determine the variables in the multi-linear model. The Bayesian Information Criterion was used to add and remove terms in the model, and to avoid data-overfitting.

## Electronic supplementary material

Supplementary Information
