# Supplementary material for: The role of city size and urban form in the surface urban heat island
Source: Sci Rep. 2017 Jul 6;7:4791. doi: 10.1038/s41598-017-04242-2 (PMC5500494; doi:10.1038/s41598-017-04242-2)
Supplement: Supplementary file 1 — Supplementary Information [file 41598_2017_4242_MOESM1_ESM.pdf]

Supplementary Information:
The role of city size and urban form in the surface urban heat island

Bin Zhou[1], **Diego Rybski**[1,*], **and Jürgen P. Kropp**[1,2]

[1]Potsdam Institute for Climate Impact Research, Potsdam, Germany
[2]Department of Geo- and Environmental Sciences, University of Potsdam, Potsdam, Germany
[*]ca-dr@rybski.de

Contents

1 Analyses on the nighttime UHI **1**
1.1 Bivariate regression 1
1.2 Multi-linear regression 1
2 Correlations between $\ln S_C$, D_f and $\ln A$ **2**
3 Linking heat transfer coefficient, area, and fractal **2**
References **3**

1 Analyses on the nighttime UHI

1.1 Bivariate regression

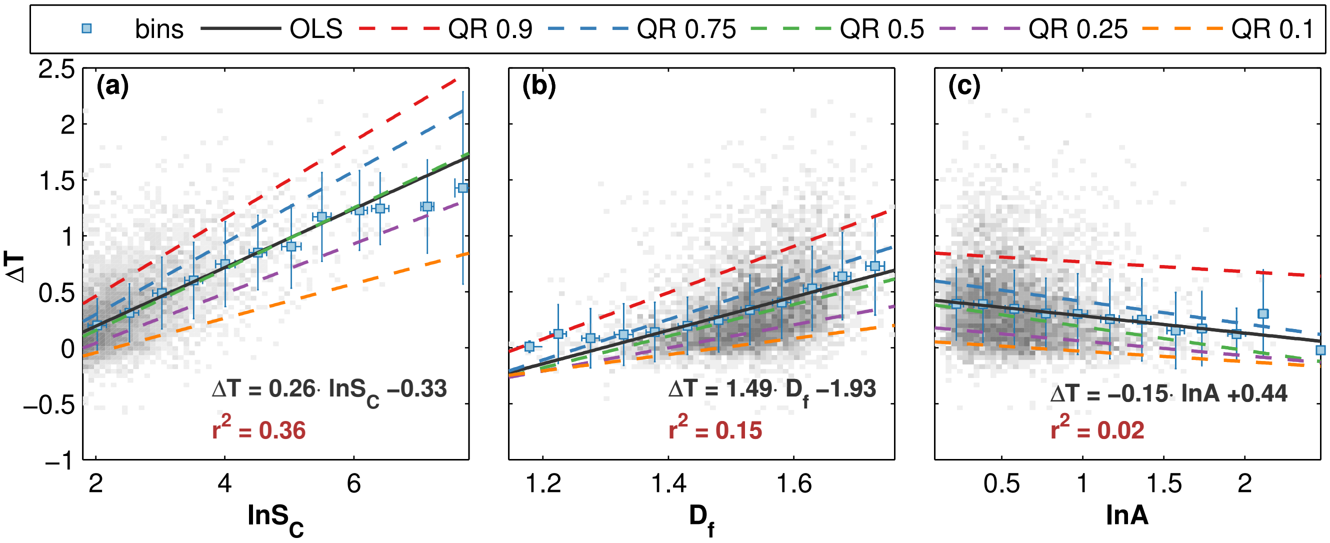

Figure S1. Nighttime surface UHI intensity (ΔT) as a function of (a) logarithm of urban cluster size $\ln S_C$, (b) fractal dimension D_f, and (c) logarithm of anisometry $\ln A$. Compared to Fig. 2 in the main text, the absolute values of regression slopes are smaller.

1.2 Multi-linear regression

The result obtained from a step-wise multi-linear regression based on 5,000 city clusters, without normalizing the dependent variables – $\ln S_C, \ln A, D_f$.

$$\Delta T = -1.03 - 0.10 \ln S_C + 0.11 D_f + 0.53 \ln A + 0.21 D_f \ln S_C - 0.33 D_f \ln A \qquad (R^2 = 0.41) \tag{1}$$

Analogous to Eq. (3) in the main text.

The relation based on normalized dependent variables

$$\Delta T = 0.33 + 0.17\,\ln S_C^* + 0.09\,D_f^* + 0.01\,\ln A^* + 0.01\,D_f^*\,\ln S_C^* - 0.01\,D_f^*\,\ln A^* \tag{2}$$

Analogous to Eq. (4) in the main text.

Since the nighttime surface UHI is generally weaker than that during daytime (which can be seen from the value range of the Y-axis), the parameters obtained from the multi-linear regression are also correspondingly smaller. However, these results are consistent with the findings in the main text, i.e. city size exerts the strongest influence ($0.17\,\ln S_C^*$), followed by fractality ($0.09\,D_f^*$), and anisometry ($0.01\,\ln A^*$).

2 Correlations between $\ln S_C$, D_f and $\ln A$

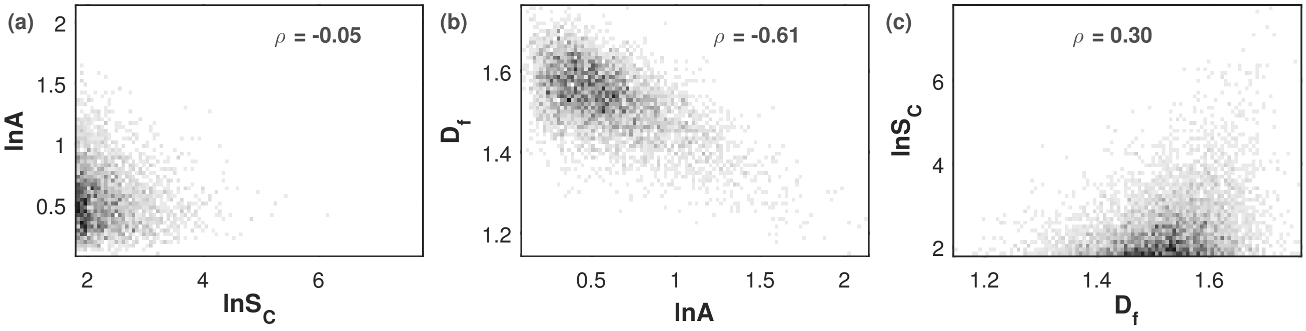

Figure S2. Correlations among intrinsic urban factors (a) $\ln A$ vs. $\ln S_C$, (b) D_f vs. $\ln A$, and (c) $\ln S_C$ vs. D_f. The grey pixels indicate the number of cities that are covered by them (the darker, the higher the density). The Pearson correlation coefficient ρ is provided in each panel.

3 Linking heat transfer coefficient, area, and fractal

Energy balance of urban surfaces

The energy balance of urban surface can be written as

$$Q^* = K\downarrow - K\uparrow + L\downarrow - L\uparrow$$
$$= Q_H + Q_E + Q_G$$

where

K short-wave radiation (arrows indicate incoming and outgoing),

L long-wave radiation (arrows indicate incoming and outgoing),

Q^* net all-wave radiation,

Q_E latent heat flux,

Q_H sensible heat flux,

Q_G conduction to or from soil.

Regardless of the anthropogenic heat release, surface temperature (T_{surface}) is mainly determined by the sensible heat flux Q_H, as $Q_H = h\Delta T = h(T_{\mathrm{surface}} - T_{\mathrm{air}})$, where h is the convection transfer coefficient. To simplify the problem, we idealize the urban surface as a flat horizontal isotropic plate, without taking into account the surface roughness. The convection heat transfer coefficient h – more precisely, how h is related to object size – is crucial to study the scale effect of the surface temperature[1].

Convection heat transfer coefficient h

The convection heat transfer coefficient h can be expressed by using the dimensionless Nusselt number Nu (the ratio of convective to conductive heat transfer),

$$h = Nu \cdot k/L \tag{3}$$

where

h convection heat transfer coefficient [W m^{-2} K^{-1}],

k thermal conductivity [W m^{-1} s^{-1}].

L characteristic length [m],

Empirically, the Nusselt number for a flat plate under turbulent forced convection can be expressed as

$$Nu = C \cdot Re^m \cdot Pr^n \tag{4}$$

where

C $\simeq 0.0296$,[2]

Re Reynolds number [dimensionless],

m ≈ 0.8 (empirical number)[2],

Pr Prandtl number [dimensionless],

n $\approx 2/3$ (empirical number)[2].

The Prandtl number is defined as the ratio of momentum diffusivity to thermal diffusivity,

$$Pr = \frac{c_p \mu}{k} \tag{5}$$

where

c_p [J kg^{-1} K^{-1}],

k thermal conductivity [W m^{-1} s^{-1}].

μ dynamic viscosity [N S m^{-2}],

The Reynolds number is defined as the ratio of inertial forces to viscous forces

$$Re = \frac{\rho v L}{\mu} \tag{6}$$

where

ρ density [kg m^{-3}],

v wind speed [m s^{-1}].

Combining Equations (3) to (6) we obtain[1]

$$\begin{aligned} &h = C k^{1-n} \rho^m \mu^{n-m} v^m c_p^n L^{m-1} \\ &h \sim L^{m-1} \quad (m-1<0). \end{aligned} \tag{7}$$

According to[3,4], the perimeter P of an object is given by the area (S) raised to the power of $\widehat{D_\mathrm{f}}/2$, i.e. $P \sim S^{\widehat{D_\mathrm{f}}/2}$, where $\widehat{D_\mathrm{f}}$ is the fractal dimension of the perimeter. Thus, the characteristic length is $L = S/P \sim S^{1-\widehat{D_\mathrm{f}}/2}$. Equation (7) can be rewritten as

$$h \sim S^{(1-\widehat{D_\mathrm{f}}/2)(m-1)} \quad (1 \le \widehat{D_\mathrm{f}} \le 2, 1-m>0). \tag{8}$$

To illustrate the influence of surface area S and fractal dimension D_f, we consider a constant influx of solar radiation. For a fixed fractal dimension, the convection transfer coefficient h decreases with increasing surface area S, resulting in a higher surface temperature. Analogously, for a fixed surface area S, the convection transfer coefficient h decreases with increasing fractal dimension D_f, resulting in a higher surface temperature.

It is worth mentioning that the fractal dimension $\widehat{D_\mathrm{f}}$ of the perimeter, also called as envelope fractal dimension, is different from the fractal dimension calculated in this study. The latter one is also known as box fractal dimension. We estimated both the envelope and box fractal dimensions by applying the box counting method to the urban outline (envelope) and urban area, respectively[4]. As shown in Fig. S3, the box fractal dimension is always larger than the envelope fractal dimension.

Analyzing our data, we obtained empirically $P \sim S^{aD_\mathrm{f}}$, with $a \approx 0.43$. Since the term $1 - aD_\mathrm{f}$ in Equation (8) is still positive, the conclusions are not affected.

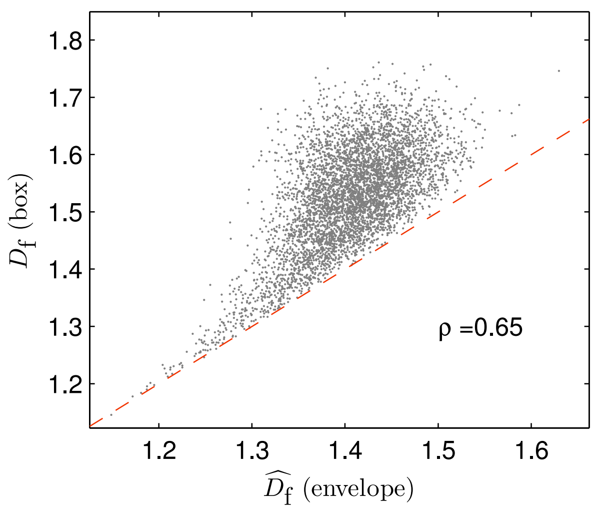

Figure S3. The box fractal dimension D_f versus the envelope fractal dimension $\widehat{D_\mathrm{f}}$. It can be seen that the box fractal dimension is always larger than the envelope fractal dimension.

References

1. Sakai, S., Iizawa, I., Onishi, M., Nakamura, M. & Kobayashi, K. Fractal Geometry of the Ground Surface and Urban Heat Island. In *Int. Conf. Urban Clim.*, July, 1–4 (2009).

2. Johnson, H. & Rubesin, M. *Aerodynamic Heating and Convective Heat Transfer* (American Society of Mechanical Engineers, 1948).

3. Lovejoy, S. Area-Perimeter Relation for Rain and Cloud Areas. *Science* **216**, 185–187 (1982).

4. Batty, M. & Longley, P. *Fractal Cities: A Geometry of Form and Function* (Academic Press Inc, San Diego, CA and London, 1994).
